# Abuse-deterrent wearable device with potential for extended delivery of opioid drugs

**DOI:** 10.1007/s13534-025-00459-7

**Published:** 2025-02-04

**Authors:** Myoung Ju Kim, Jae Min Park, Jun Su Lee, Ji Yang Lee, Juhui Lee, Chang Hee Min, Min Ji Kim, Jae Hoon Han, Eun Jung Kwon, Young Bin Choy

**Affiliations:** 1 Interdisciplinary Program in Bioengineering, College of Engineering, Seoul National University, Seoul, 08826 Republic of Korea; 2 Integrated Major in Innovative Medical Science, College of Medicine, Seoul National University, Seoul, 08826 Republic of Korea; 3 Department of Medicine, Seoul National University College of Medicine, Seoul, 03080 Republic of Korea; 4 Institute of Medical and Biological Engineering, Medical Research Center, Seoul National University, Seoul, 03080 Republic of Korea; 5 Department of Biomedical Engineering, Seoul National University College of Medicine, Seoul, 03080 Republic of Korea; 6 Innovative Medical Technology Research Institute, Seoul National University Hospital, Seoul, 03122 Republic of Korea; 7 ToBIOS Inc., 3F, 9-7 Seongbuk-ro 5-gil, Seongbuk-gu, Seoul, 02880 Republic of Korea

**Keywords:** Abuse-deterrent, Opioid drugs, Sustained drug delivery, Wearable device

## Abstract

**Purpose:**

Unethical attempts to misuse and overdose opioids have led to strict prescription limits, necessitating frequent hospital visits and prescriptions for long-term severe pain management. Therefore, this study aimed to develop a prototype wearable device that facilitates the extended delivery of opioid drugs while incorporating abuse-deterrent functionality, referred to as the abuse deterrent device (ADD).

**Methods:**

The ADD was designed and fabricated using 3D-printed components, including reservoirs for the drug and contaminant, as well as an actuator. In vitro tests were conducted using a skin-mimicking layer and phosphate-buffered saline (PBS) to evaluate the drug release profile and the effectiveness of the ADD abuse-deterrent mechanism.

**Results:**

Under simulated skin attachment, ADD demonstrated sustained drug release with the potential to persist for up to 200 days. Upon detachment from the skin mimic, the mechanical components of the ADD facilitated immediate exposure of the contaminant to the drug and effectively halted further drug exposure throughout-diffusion.

**Conclusion:**

Wearable ADD provides a secure and practical solution for the long-term treatment of high-risk medications such as opioids, enhances patient convenience, and addresses important public health concerns.

**Supplementary Information:**

The online version contains supplementary material available at 10.1007/s13534-025-00459-7.

## Introduction

Severe pain, often resulting from late-stage cancer, treatment side effects, or major surgeries, significantly impairs the daily function of an individual and often necessitates the use of strong opioids for adequate relief [1–3]. However, opioid-based analgesics present multifaceted issues, including opioid use disorder (OUD) [4], which is characterized by opioid dependence and addiction [5–7], and critical social issues, such as the unauthorized distribution of opioids to the black market [8, 9]. Owing to the illegal acquisition of many opioids and the rise in drug overdose deaths, which reached more than 80,000 in 2021 [10], stricter prescribing limits for healthcare professionals became necessary. Therefore, for long-term treatment, patients need more frequent hospital visits, and clinicians need to issue prescriptions more frequently [11].

To prevent opioid abuse, abuse deterrent formulations (ADFs), which generally incorporate both opioids and antagonists, have been developed [12]. These formulations exploit the differences in the bioavailability of agonists and antagonists, depending on the administration route. Thus, the opioid was absorbed better than its antagonist when taken as prescribed (e.g., orally), whereas the antagonist was absorbed more easily than the opioid when administered by non-prescribed routes (e.g., injected or inhaled), thereby reducing or eliminating the efficacy of the opioid [13]. However, this deterrent strategy is fundamentally administration route dependent; thus, a holder may still take an excessive amount of drug beyond what is prescribed or safe [14]. Consequently, social issues related to unethical opioid distribution may remain unresolved, necessitating dose-limiting prescriptions through regulations that require frequent patient hospital visits and more frequent prescriptions by clinicians.

Therefore, in this study, we propose a wearable device equipped with abuse-deterrent functionality (i.e., abuse deterrent device (ADD)). The ADD herein incorporates two distinguished key features, which involve sustained drug release and abuse-deterrent functionality. When the ADD is attached to skin, it enables continuous drug release at a predetermined rate, ensuring consistent dosing and preventing overdosing. However, if an attempt is made to detach the ADD, it automatically contaminates the remaining drug and ceases drug release. This design aims to discourage unethical attempts to remove drugs from the ADD. With this safeguard configuration, ADD can contain a reservoir of therapeutic opioids that can provide a comparable amount needed for long-term treatment, thereby extending therapy with a single prescription.

In this study, we designed an ADD without electronics to prioritize simplicity, reliability, and potentially lower production costs. To maintain the abuse-deterrent functionality, the ADD operated based on mechanical actuation principles, which varies depending on whether the device was attached or detached from the skin. ADD primarily consists of a drug reservoir adjacent to a contaminant reservoir separated by a thin membrane. Within this straightforward configuration, a piston was installed in the ADD, which moves forward in steps by releasing the pins upon skin attachment and detachment. For skin attachment, a needle unit was installed on the ADD and inserted into the skin. The onset pin was then released to advance the piston, filling space between empty needle and the drug reservoir with drug and initiating drug release. Upon skin detachment, the security pin was automatically released to cause the piston to move further, plugging the needle to stop the drug release and tearing the membrane to mix the drug with the contaminant.

To assess the proof-of-principle design, the ADD in this study was fabricated by assembling each constituent unit prepared using 3D printing. The assembled ADD was loaded with fluorescein as a model drug and rhodamine B as a model contaminant. To test the drug-release profiles, the ADD was attached to an artificial skin layer. Next to the skin layer, a chamber filled with simulated biological fluid (phosphate-buffered saline (PBS) at pH 7.4) served as the medium for drug release, with the needle inserted into it. Under these conditions, we examined drug release profiles upon skin attachment scenario. Additionally, we intentionally detached the ADD to examine the drug release behavior, and identify the actuator’s operation followed by membrane rupture and drug contamination.

## Materials and methods

### Materials

Veroclear and SUP706 were purchased from Stratasys (Rehovot, Israel) for the 3D printing and supporting materials, respectively. Stainless-steel springs were purchased from Springfarm (Chuncheon, South Korea). The septum was obtained from a screw cap purchased from Futects (Daejeon, South Korea). The rubber stopper was obtained from a 1 ml syringe purchased from Koreavaccine (Seoul, Korea). The 32G needle was purchased from Becton Dickinson (Seoul, Korea). Fluorescein sodium salt and Rhodamine B were purchased from Sigma-Aldrich (St. Louis, MO, USA). Medical epoxy was purchased from Epoxy Technology (MA, USA). Ecoflex 00-10 was purchased from Smooth-On (PA). Circular Nd magnets were purchased from JL Magnet (Seoul, South Korea). Membranes were purchased from Korea Industry (Iksan, Korea). The polyurethane film was purchased from Nihon Matai (Tokyo, Japan). Double-sided adhesive tape was purchased from Tesa (Seoul, South Korea).

### ADD fabrication

**Fig. 1:**
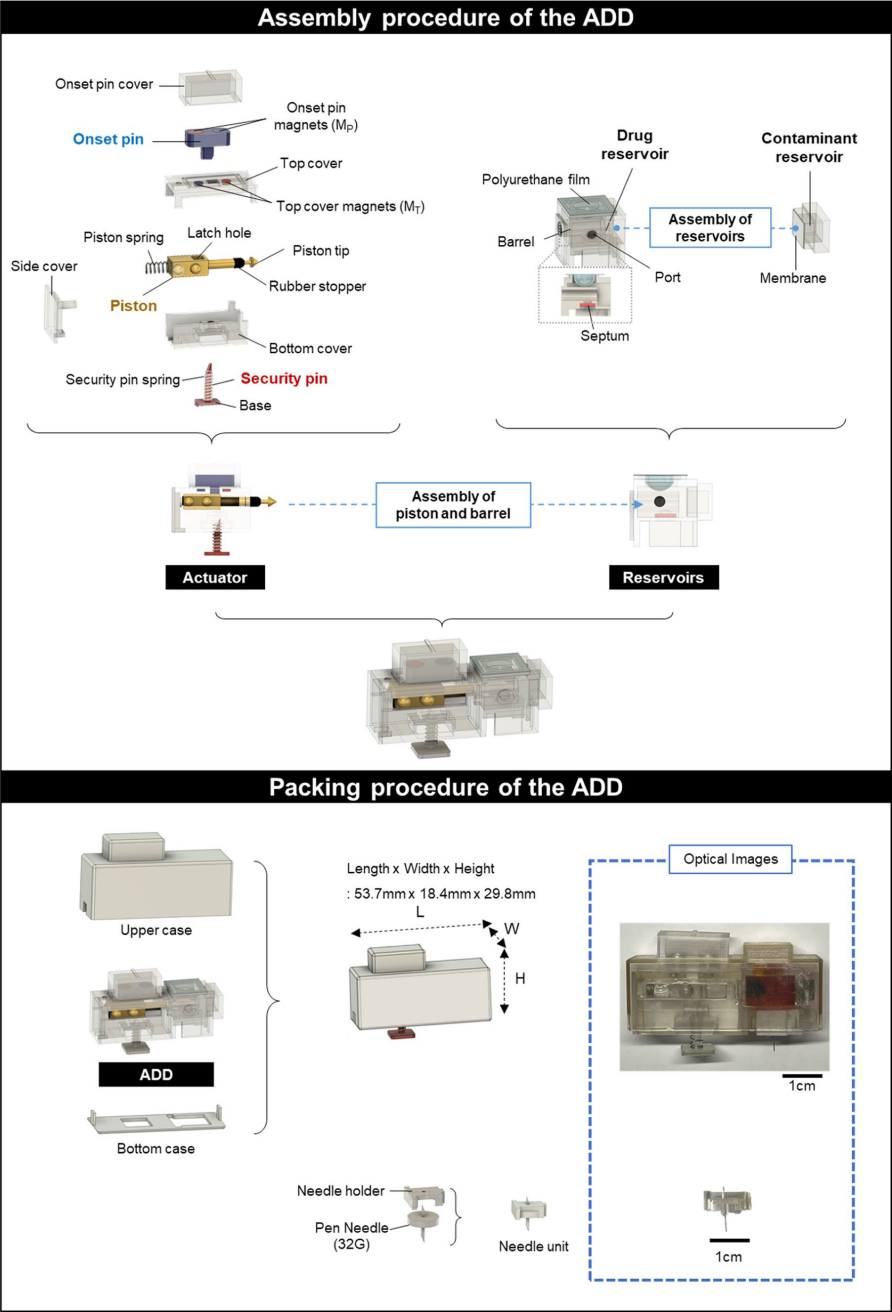
Schematic description depicting assembly and packing procedure of the abuse-deterrent device (ADD). **a** Assembly procedure of the ADD and **b** Packing procedure of the ADD

Except for the commercially obtained elements, we designed all the constituent units using computer-aided design software (Fusion 360, Autodesk, CA, USA), manufactured them using a 3D printer (Objet 30 Pro; Stratasys, Rehovot, Israel), and assembled the manufactured units with medical epoxy to fabricate the ADD. As shown in Fig. 1, the ADD comprised two major parts: reservoirs and actuators. The device consisted of two distinct reservoirs: one for the model drug, fluorescein, and the other for the model contaminant, rhodamine B. The drug reservoir was a barrel sealed at the top with an elastic polyurethane (PU) film and at the bottom with a septum. The contaminant reservoir, which contained a fine rhodamine B powder (10 mg), was sealed using a membrane. The drug and contaminant reservoirs were then combined, where those two reservoirs were separated by the membrane. The actuator assembly featured a piston equipped with a piston tip, rubber stopper, latch hole, and piston spring covered by top, bottom, and side covers. During the assembly, the piston spring was compressed against the side cover and the onset pin was inserted into the top cover and latch hole to secure the piston. The onset pin contained two magnets with alternating polarities aligned with opposite-polarity magnets in the top cover, allowing stable positioning through magnetic attraction [15]. A security pin with a base to maintain stable skin contact was aligned through a hole in the bottom cover and inserted into the latch hole of the piston. The piston tip was then inserted into the barrel of the drug reservoir, and the actuator and reservoir were seamlessly bonded. The drug reservoir was then filled with 1 ml of fluorescein solution (10 mg/ml) prepared in phosphate-buffered saline (PBS, pH 7.4) via a port sealed with a lid. The assembly was securely enclosed in the upper and lower cases. The needle unit was prepared separately with a needle (32 G), which was installed on the ADD by inserting one end of the needle through the septum in the drug reservoir upon skin attachment.

### In vitro performance test

To examine in vitro drug release profiles, we used a skin-mimicking layer made of Ecoflex 00–10 [16, 17]. In this experiment, a needle unit was installed on the ADD. The ADD was firmly attached to a skin-mimicking layer, allowing the security pin to be pushed and engaged with the ADD. In this setup, the needle was inserted into a separate chamber filled with PBS (pH 7.4) to ensure that the needle tip was completely immersed in the PBS (Online Resource 1). Then, the onset pin was disengaged using an onset key, initiating drug release at day 0. Specifically, 1 ml of the medium was extracted and the same volume of fresh medium was added daily for 7 days. The collected medium was assayed spectrophotometrically using a UV-Vis spectrophotometer (UV-1800, Shimadzu, Kyoto, Japan) at an absorption wavelength of 460 nm to determine the amount of fluorescein released.

To validate the abuse-deterrent functionality, the drug reservoir in the ADD was replaced with 1 ml of PBS (pH 7.4) without the drug and applied to the skin-mimicking layer, as described above. We intentionally detached the ADD and visually observed the drug reservoir to assess whether the contaminant mixed with the drug reservoir during detachment. Next, we reattached the ADD to the layer with the needle inserted, and the release medium was collected daily for 7 days to assess whether any leakage of the contaminant occurred. The collected liquid and medium were analyzed using a UV-Vis spectrophotometer at an absorption wavelength of 554 nm to detect the presence of the model contaminant rhodamine B.

## Results

### Working principle

**Fig. 2:**
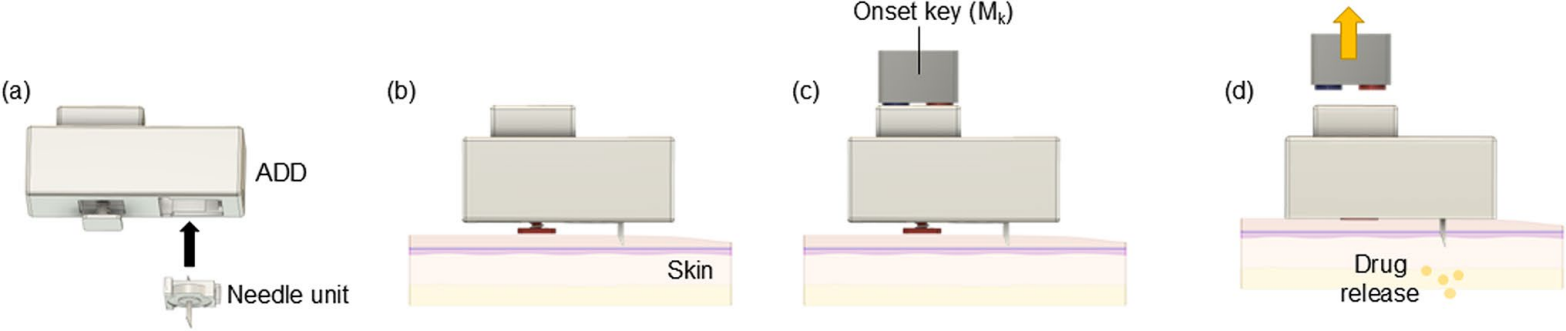
Schematic description of the user guide for the skin attachment of the ADD. **a** Install the needle unit to the ADD through the septum. **b** Attach the ADD to skin by inserting the other end of the needle into skin and also pushing the security pin into the ADD. **c** Use the onset key to pull the onset pin and **d** initiate drug release

We aimed to fabricate an ADD prototype that can deliver a drug in a sustained manner while attached to the skin, however, when detached, stops delivery and contaminates the drug. This design allows the ADD to support long-term therapy while minimizing the risk of unethical attempts to remove the drug from the device. Figure 2 illustrates a schematic of the user guide for the ADD skin attachment. Before skin attachment, a needle unit was installed in the ADD by inserting one end of the needle through the septum into the drug reservoir. The ADD was attached to the skin by inserting the other end of the needle into the skin and pushing the security pin until it was completely engaged in the ADD. Subsequently, the onset key, which contained two magnets (M_K_) with alternating polarities opposing those of the onset pin magnets (M_P_), was brought close to pull the onset pin and initiate drug release.

**Fig. 3:**
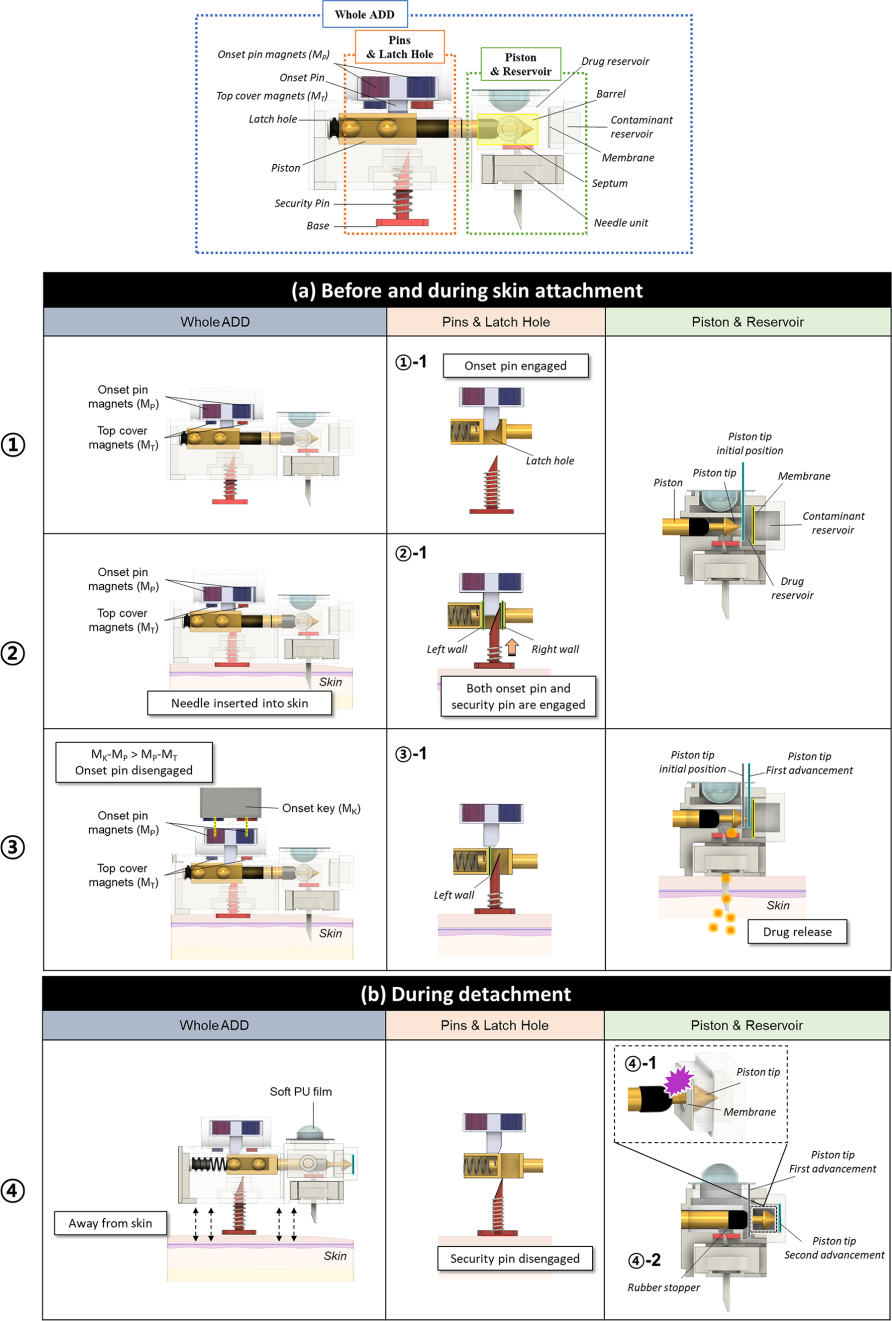
Working principle of the ADD, **a** before and during skin attachment and **b** during skin detachment

Figure 3a illustrates the detailed working principle of the ADD before and while attached to the skin. While the ADD is not in use, the piston is immobilized by the onset pin (①-1) positioned within the latch hole and held securely in place by a magnetic attraction between the top cover magnets (M_T_) and the onset pin magnets (M_P_) with opposite polarities. Immediately before the skin attachment, a needle unit was installed on the ADD. At this stage, the liquid drug solution did not flow out, because the air inside the needle acted as a plug. When the ADD is applied to the skin, the needle is inserted into the subcutaneous space, and the bottom case is firmly attached to the skin using double-sided adhesive tape. Therefore, this action also pushes the base of the security pin, compressing the security pin spring and allowing the security pin to be inserted into the latch hole in the piston (②). In this state, the onset pin and the security pin are engaged into the left and right walls in the identical latch hole, respectively, and thus, the onset pin restrains the force exerted by the compressed piston spring (②-1). We employed an onset key with two magnets (M_K_) of alternating polarity to initiate drug release. For this, the M_K_ were brought close and aligned with the M_P_ of opposite polarities (③). This allowed the onset pin to move upward and be disengaged from the latch hole in the piston, as we intentionally utilized a stronger magnetic force between M_K_ and M_P_ than between M_P_ and M_T_ (③-1). When the onset pin holding the left wall of the latch hole was removed, the piston was pushed by the piston spring and moved slightly to the right (approximately 3 mm), causing the security pin to engage with the left wall of the latch hole. During this transition, the piston created a slightly positive pressure in the drug reservoir, pushing air out of the needle. This made the conduit inside the needle continuous with the drug solution, allowing for drug diffusion through the needle (③-2). When the onset key with M_K_ was removed, the attraction force between M_T_ and M_P_ moved the onset pin downward; however, the pin could not be inserted into the latch hole owing to the displacement already made by the piston.

Figure 3b illustrates the working principle of ADD when skin detachment is attempted. At this stage, the compressed security pin spring was released, disengaging the security pin from the latch hole in the piston. This rapidly released the compressed piston spring, causing the piston to move further to the right. The piston tip then reached and teared the membrane, sealing the contaminant reservoir and exposing it to the drug reservoir (④-1). This movement of the piston also blocked the path between the barrel and the needle with a rubber stopper, thereby stopping the out-diffusion of the contaminated drug solution (④-2). During this transition, the soft PU film at the top of the drug reservoir became elongated and convex upward, compensating for the slightly positive pressure created by the advancement of the piston in the barrel of the drug reservoir.

### In vitro performance test

**Fig. 4:**
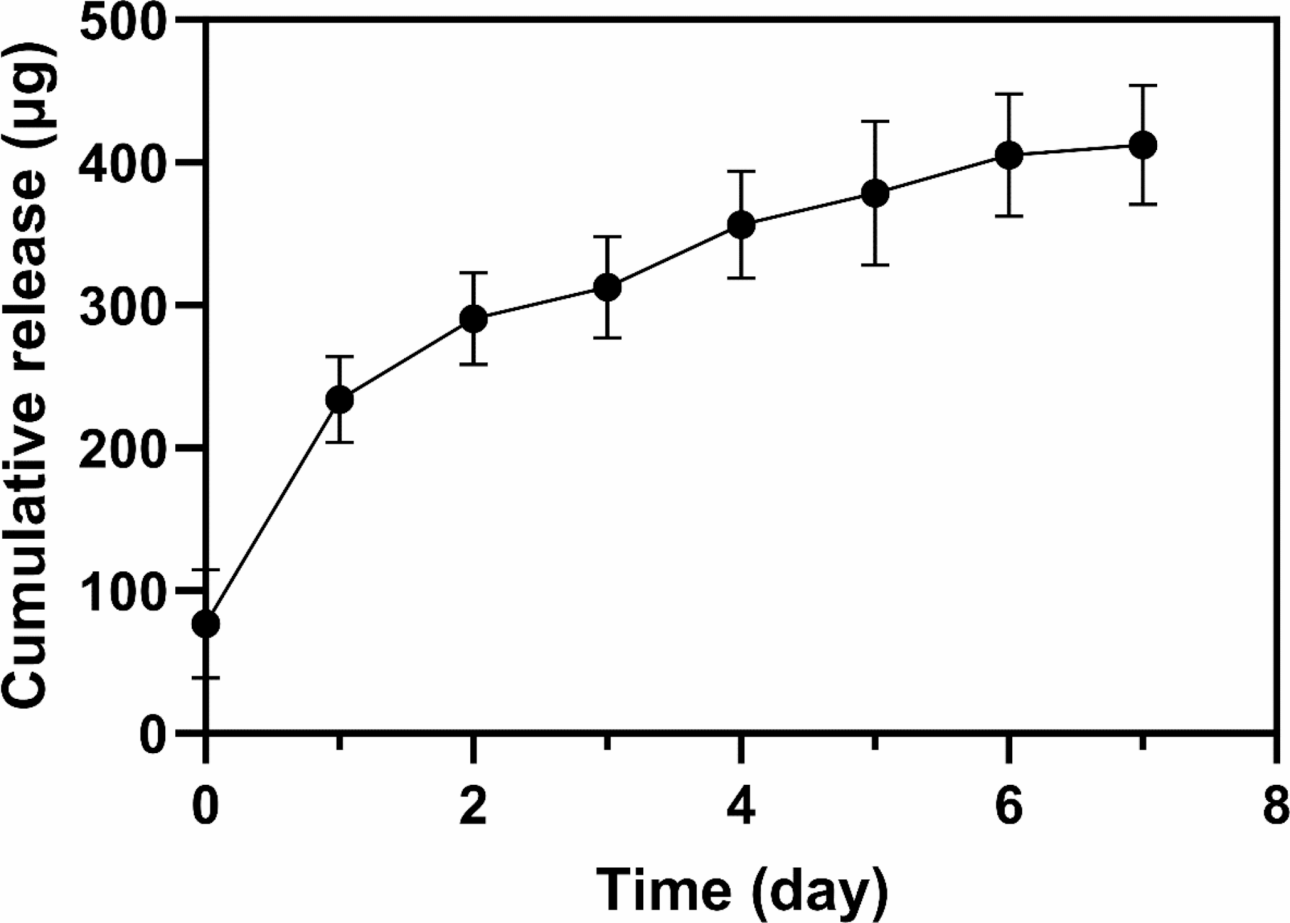
In vitro drug release profile of the add attached on a skin-mimicking layer with the needle immersed in the ph 7.4 pbs filled in the neighboring chamber. The release was initiated by pulling the onset pin with the onset key

To examine the drug release profile upon ADD skin attachment, we used a skin-mimicking layer and a bath filled with PBS (pH 7.4) to simulate the skin layer and interstitial fluid in the subcutaneous space (Online Resource 1). Drug release was initiated after the onset pin was disengaged using the M_K_ of the onset key aligned to the M_P_ of the onset pin (Online Resource 2). With a single polarity, the onset pin could not be disengaged, even with a strong magnet (Online Resource 3). As shown in Fig. 4, although small, a burst release occurred immediately after disengaging the onset pin at day 0, as some drug solution appeared to be pushed out through the needle during piston advancement. However, afterward, the drug was released in a sustained manner at a rate of about 48 µg per day. For 7 days, the ADD herein released about 410 µg drug, which accounted for about 4% of the total loading amount. When extrapolated, this would allow for more than 200 days of drug release from a 1 ml drug reservoir of a single ADD herein.

**Fig. 5:**
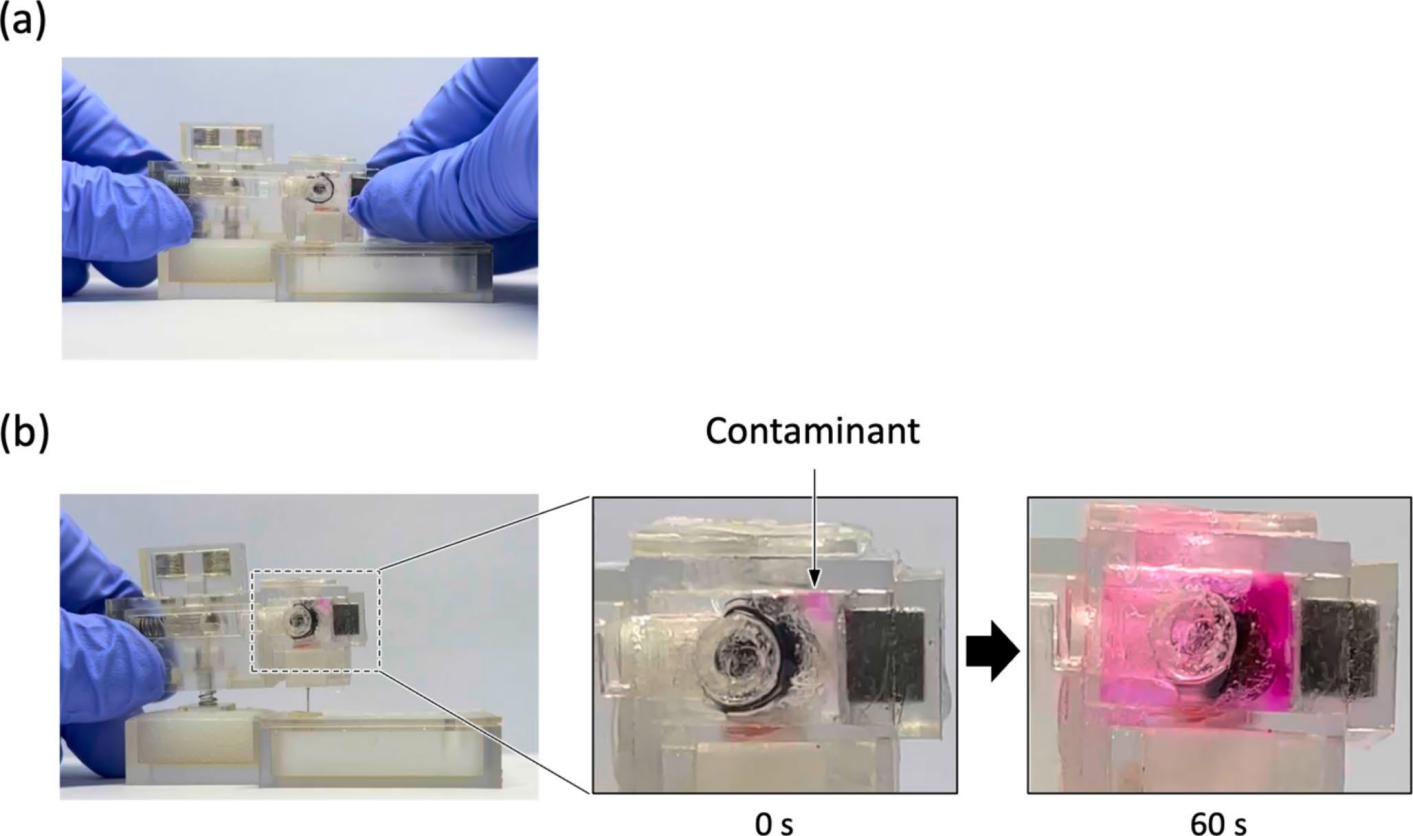
Optical images of the ADD during simulated conditions of (**a**) skin attachment and (**b**) skin detachment. After detachment, the red contaminant was readily exposed to the drug reservoir

**Fig. 6:**
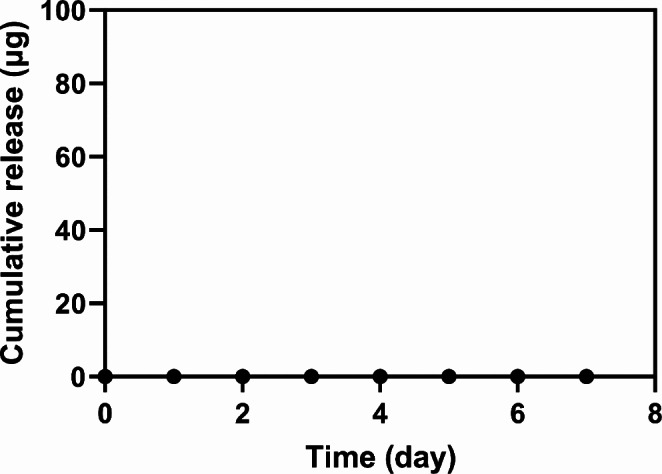
In vitro release profile of the contaminant. Right after skin detachment, the ADD was re-attached to the skin-mimicking layer and PBS chamber

To assess the abuse-deterrent capacity, we applied ADD filled with PBS without fluorescein to the skin-mimicking layer, as described above, and intentionally detached it. Upon detachment of the ADD, the security pin immediately escaped from the latch hole in the piston, causing it to advance and rupture the contaminant reservoir membrane. Consequently, the model contaminant, rhodamine B, was exposed to the drug reservoir (Fig. 5). We also aimed to further evaluate the safety of the ADD in cases where it was reapplied to the skin even after detachment. In our experimental setup, when the detached ADD was reapplied to the skin, almost no release of rhodamine B was observed (Fig. 6). This suggests that the bodily fluid would not be exposed to the contaminant in the design of the ADD. The rubber stopper in the piston properly sealed the barrel and needle from the drug reservoir, preventing the out-diffusion of the contaminated compounds.

## Discussion

Opioid analgesics are effective for managing severe pain, such as that associated with cancer or acute conditions. Previously, wearable devices for on-demand drug delivery have been recognized for providing patients the convenience of self-administration of medications [18]. However, their use with opioid analgesics has been limited due to significant risks of misuse, which can result in life-threatening and ethically concerning situations [19]. For example, wearable drug delivery devices for fentanyl are strictly available only in hospital settings under the Risk Evaluation and Mitigation Strategy (REMS) program, due to the high potential for abuse [20]. These concerns often require stricter prescription limits, which can be inconvenient for patients as more frequent hospital visits and prescriptions will be required [21].

Therefore, we developed ADD, a proof-of-principle wearable device that combines sustained drug release with abuse-deterrent functionality. ADD is easy to use and is attached to the skin via needle insertion to deliver medication subcutaneously over an extended period. Crucially, an ADD is designed to automatically halt the drug release and contaminate the drug in the reservoir if the user attempts premature removal. This ensures the primary advantage of continuous drug release, while preventing the user from altering the dosage or misusing the drug. Consequently, the ADD can facilitate a single prescription for long-term opioid analgesics. In our ADD, the rate of drug release was predetermined by the needle size and drug concentration, which served as the diffusion barrier and depot conditions, respectively. These parameters can be modified or customized during production to adjust the drug dosage for individual patients. The size of the ADD is comparable to that of commercially available wearable drug delivery devices [22, 23]. However, it operates entirely through mechanical modalities, without electronics, allowing for a more compact design. Therefore, by optimizing the drug load and concentration based on prescription requirements, ADD can further enhance patient convenience [24].

For added security, the magnets controlling the onset pin can be specially patterned, similar to a sophisticated key, to ensure that only authorized personnel can initiate drug release [15, 25]. Additionally, an external casing may need to be constructed from rigid metallic materials to prevent attempts to extract the drug while the ADD is attached to the skin. Model drugs and contaminants were used to evaluate the feasibility of the prototype. To achieve practical application, the efficacy and safety of ADD must be validated using actual opioid analgesics.

## Conclusion

In this study, we have presented a wearable drug delivery device designed for continuous medication administration that features robust abuse-deterrent functionality to ensure the safe and extended delivery of opioid drugs. Upon attachment to the skin, drug release was initiated only when the designated personnel removed the onset pin. Once activated, the drug release rate remained fixed and unalterable. In the event of detachment, a contaminant within the device neutralized the efficacy of the drug while keeping it contained without exposure. Therefore, this abuse-deterrent wearable device offers an effective solution for managing high-risk medications, such as opioids, enhancing patient convenience, and addressing crucial public health concerns.

## Electronic supplementary material

Below is the link to the electronic supplementary material.

Supplementary Material 1

Supplementary Material 2

Supplementary Material 3
