# Supplementary material for: Abuse-deterrent wearable device with potential for extended delivery of opioid drugs
Source: Biomed Eng Lett. 2025 Feb 4;15(2):427–35. doi: 10.1007/s13534-025-00459-7 (PMC11871182; doi:10.1007/s13534-025-00459-7)
Supplement: Supplementary file 3 — Supplementary Material 3 [file 13534_2025_459_MOESM3_ESM.pdf]

Biomedical Engineering Letters

Abuse-deterrent Wearable Device with Potential for Extended Delivery of Opioid Drugs

Myoung Ju Kim[1,2]†, Jae Min Park[3]†, Jun Su Lee[3]†, Ji Yang Lee[3]†, Juhui Lee[1,2]†, Chang Hee Min[1], Min Ji Kim[1], Jae Hoon Han[1], Eun Jung Kwon[1,2], Young Bin Choy[1,2,4,5,6,7]*

[1] Interdisciplinary Program in Bioengineering, College of Engineering, Seoul National University; Seoul 08826, Republic of Korea

[2] Integrated Major in Innovative Medical Science, College of Medicine, Seoul National University; Seoul 08826, Republic of Korea

[3] Department of Medicine, Seoul National University College of Medicine; Seoul 03080, Republic of Korea

[4] Institute of Medical and Biological Engineering, Medical Research Center, Seoul National University; Seoul 03080, Republic of Korea

[5] Department of Biomedical Engineering, Seoul National University College of Medicine; Seoul 03080, Republic of Korea

[6] Innovative Medical Technology Research Institute, Seoul National University Hospital; Seoul 03122, Republic of Korea

[7] ToBIOS Inc., 3F, 9-7 Seongbuk-ro 5-gil, Seongbuk-gu, Seoul 02880, Republic of Korea

† These authors contributed equally to this work.

*Corresponding author: Young Bin Choy

Email: ybchoy@snu.ac.kr

This PDF file includes:

Online Resource 1

(a)

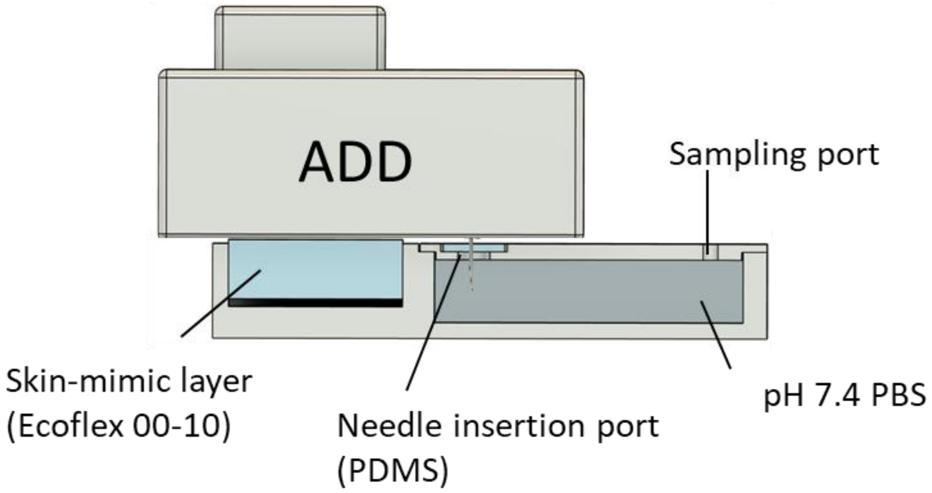

(b)

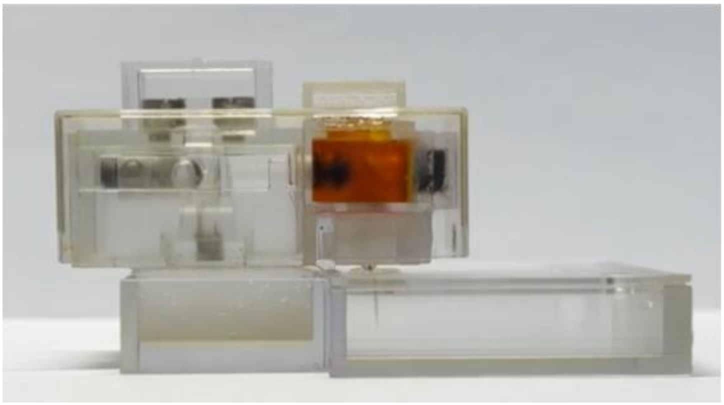

Online Resource 1 Experimental setup for in vitro tests: (a) Schematic and (b) Optical image. The security pin of the ADD is positioned on a skin-mimic layer (Ecoflex 00-10), while the tip of the needle penetrates a PDMS-based insertion port that represents the skin layer and is immersed in pH 7.4 PBS, simulating interstitial fluid
